# No strategy to meet the HCV epidemic

**DOI:** 10.1186/1471-2334-14-S6-S2

**Published:** 2014-09-19

**Authors:** Olav Dalgard, Stefan Mauss

**Affiliations:** 1 Department of Infectious Diseases, Akershus University Hospital, Lørenskog Norway; 2 Center for HIV and Hepatogastroenterology, Duesseldorf Germany

## 

Despite an estimated 84,000 deaths related to hepatitis C virus (HCV) infection in Europe each year, no strategy to respond to HCV is in place in the majority of European countries according to two surveys published in *BMC *[1-3]. Why is this so and does it matter?

The lack of public interest in this important threat to public health is striking and probably explains the lack of policy responses. Hepatitis is correctly described as the silent epidemic in which patients carry the virus for decades before a quarter succumb to liver failure or liver cancer [4,5]. Most patients acquiring hepatitis C in recent decades in Europe have been infected through injecting drug use [6]. The disease therefore carries an important stigma discouraging individuals from publicly acknowledging their infection; this stigma probably also explains why patient groups are conspicuously absent. Furthermore, successful HCV treatment lacks “the Lazarus effect” seen after successful treatment of HIV. By the time symptoms of liver failure appear, HCV treatment has until recently been unable to reverse the disease. Treatment regimens have long been associated with considerable toxicity limiting access to therapy due to medical, social and structural reasons.

But this scenario is rapidly changing. A paradigm shift is underway, with combinations of direct-acting antivirals replacing interferon-based therapies [7]. Treating almost every person with HCV regardless of liver disease stage, viral genotype, past therapies or comorbidities is possible. Hard-to-treat populations such as people who inject drugs, HIV-coinfected individuals and patients with psychiatric comorbidities will be much easier to treat with high cure rates. However, per-patient treatment costs will also increase and will be a challenge for national health systems due to the expanding number of patients. This will be a substantial challenge in particular for Southern and Eastern Europe, including Russia, where HCV prevalence and incidence rates are high. Even in western European countries, however, the current pricing of new direct-acting antivirals enabling interferon-free therapy – such as sofosbuvir and simeprevir – is threatening to affect healthcare budgets substantially. As a consequence, access to this beneficial therapy will be sadly limited due to conflicting economic interests.

For this reason, the lack of a strategy to fight HCV is a matter of concern. In the meantime the high prices of the new drugs may prove to have the paradoxical effect of increasing public awareness of this disease, motivating policy-makers to put in place good strategies for hepatitis C prevention and treatment and bring down prices.

## Competing interests

This article is endorsed by the European Association for the Study of the Liver; this text, however, represents the personal perspective of Olav Dalgard and Stefan Mauss, and they declare that they have no competing interests.

## Declarations

This article has been published as part of *BMC Infectious Diseases* Volume 14 Supplement 6, 2014: Viral Hepatitis in Europe. The full contents of the supplement are available online at http://www.biomedcentral.com/bmcinfectdis/supplements/14/S6. The publication charges for this supplement were funded by AbbVie as an unrestricted grant to Rigshospitalet, the University of Copenhagen. AbbVie further funded the printing of the supplement with additional financial support from the Drug Prevention and Information Programme (DPIP) of the European Union.
